# Prolonged exposure to multi-walled carbon nanotubes dysregulates intestinal *mir*-*35* and its direct target MAB-3 in nematode *Caenorhabditis elegans*

**DOI:** 10.1038/s41598-019-48646-8

**Published:** 2019-08-21

**Authors:** Yunli Zhao, Ling Jin, Yuan Wang, Yan Kong, Dayong Wang

**Affiliations:** 1 Department of Preventive Medicine, Bengbu Medical College, Bengbu, 233030 China; 2 Medical School, Southeast University, Nanjing, 210009 China

**Keywords:** Environmental impact, Biomarkers

## Abstract

In nematode *Caenorhabditis elegans*, some microRNAs (miRNAs) could be dysregulated by multi-walled carbon nanotubes (MWCNTs), suggesting their involvement in regulating the response of nematodes to MWCNTs. Among these dysregulated miRNAs induced by MWCNT exposure, prolonged exposure to MWCNTs increased *mir*-*35* expression. *mir*-*35* further acted in the intestine to regulate the response to MWCNTs. In the intestine, a transcription factor MAB-3 was identified as its target in regulating the response to MWCNTs. Moreover, during the control of response to MWCNTs, MAB-3 acted upstream of DAF-16, a fork head transcriptional factor in insulin signaling pathway. Therefore, MWCNTs exposure potentially dysregulates intestinal *mir*-*35* and its direct target MAB-3, which may activate a protective intestinal response of nematodes against the MWCNTs toxicity.

## Introduction

During the last decades, carbon nanotubes (CNTs) have attracted the great interest for some of their unique properties, such as stability, rigidity, extraordinary tensile strength, and efficient heat conduction^1–3^. Multi-walled CNTs (MWCNTs) consisting of concentric layers of single-walled CNTs have the potential in biomedical areas, including drug delivery, biosensors, medical imaging, and targeted therapeutic^4–6^. Meanwhile, safety and exposure concerns of MWCNT have aroused with its wide application. Some works have been performed with the aim to elucidate the cellular and the molecular mechanisms of MWCNTs toxicity in organism^7–10^. Nevertheless, the underlying molecular mechanisms for the response of organisms to MWCNTs are still largely unclear.

MicroRNAs (miRNAs), small noncoding regulatory molecules, regulate the expression and the functions of their target messenger RNAs (mRNAs) by binding to certain sites in 3′ untranslated region (UTR) and affecting their translation into proteins^11^. It has been well known that one certain miRNA can simultaneously target several or many mRNAs^12^. Some miRNAs have been shown to play pivotal roles in regulating various biological processes, such as growth and development^13,14^. The increasing evidence has further suggested that some miRNAs may function in the control of stress response^15,16^.

Nematode *Caenorhabditis elegans* is an important model animal for the identification and the functional analysis of miRNAs^13,15^. Meanwhile, *C*. *elegans* is very sensitive to the toxicity of environmental toxicants, and suitable for the elucidation of molecular mechanism for the observed toxicity of environmental toxicants at the whole animal level^17^. The previous studies have demonstrated that some miRNAs could be dysregulated by carbon-based nanomaterials, such as MWCNTs and graphene oxide (GO), and some miRNAs were further shown to be required for the control of toxicity induction of MWCNTs or GO^18,19^.

Among the dysregulated miRNAs by MWCNTs exposure, *mir*-*35* could be increased by prolonged exposure to MWCNTs at concentrations ≥100 μg/L^18^. In addition, mutation of *mir*-*35* could induce a susceptibility to MWCNTs toxicity at various aspects, such as induction of intestinal reactive oxygen species (ROS) and decrease in locomotion behavior^18^. However, the underlying mechanism for the role of *mir*-*35* in regulating the response to MWCNTs is still unknown in nematodes. In this study, we first examined the tissue-specific activity of *mir*-*35* in regulating the response to MWCNTs. Moreover, we identified the target of intestinal *mir*-*35* and the underlying mechanism for intestinal *mir*-*35* in regulating the response to MWCNTs. Our results demonstrated that the increase in *mir*-*35* expression mediated a protective intestinal response to MWCNTs by suppressing function of MAB-3-DAF-16 signaling cascade. Our data provides an important molecular basis for intestinal response to MWCNTs in organisms.

## Results

### Alteration in *mir*-*35* expression in MWCNTs exposed nematodes

After prolonged exposure from L1-larvae to adult day-1, MWCNTs at concentrations more than 0.1 μg/L significantly increased the *mir*-*35* expression (Fig. S1). Meanwhile, in the isolated intestine, MWCNTs (≥0.1 μg/L) also significantly increased the *mir*-*35* expression (data not shown). Additionally, the *mir*-*35* expression was concentration-dependent in MWCNTs exposed nematodes (Fig. S1).

### Tissue-specific activity of *mir*-*35* in regulating the response to MWCNTs

**Figure 1:**
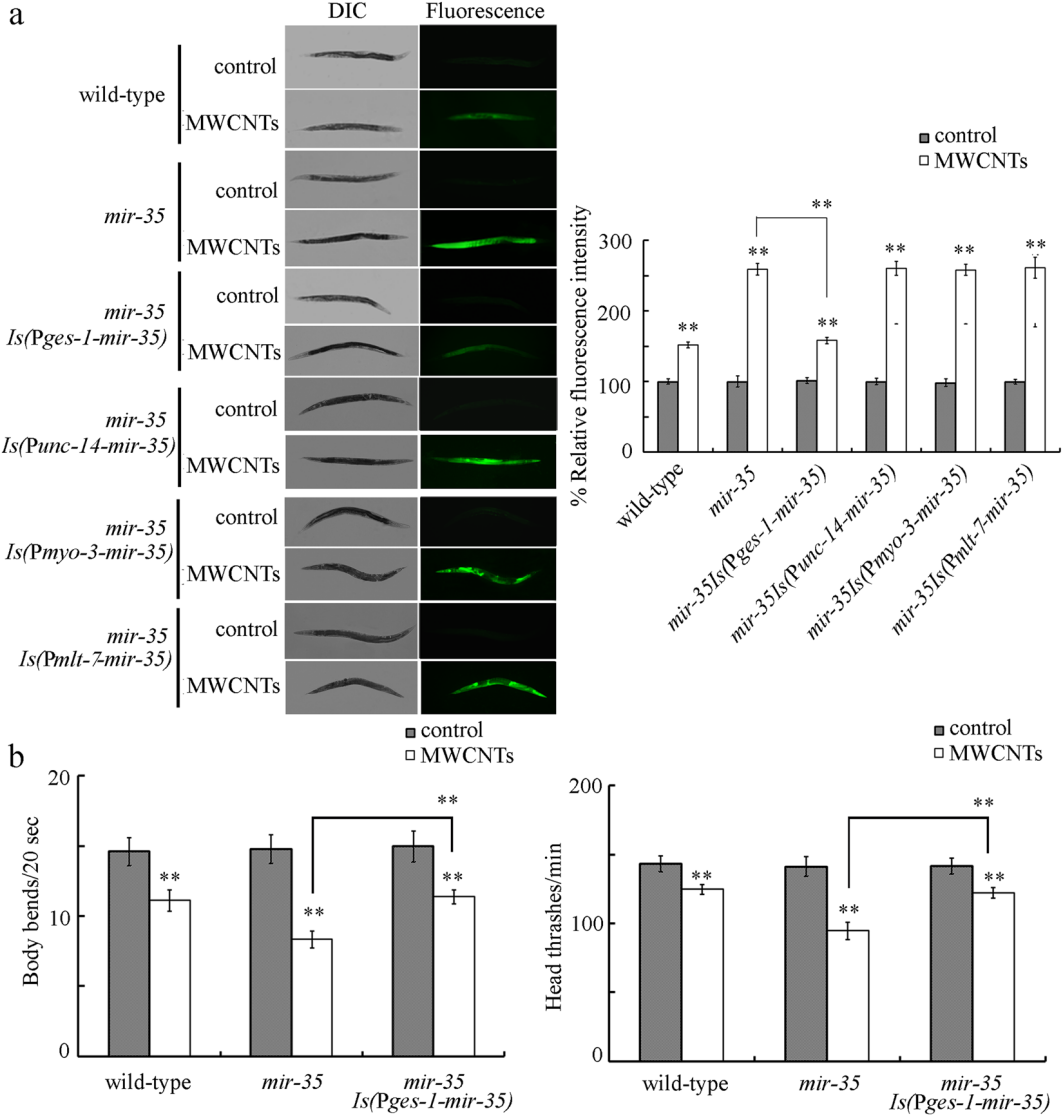
Tissue-specific activity of *mir*-*35* in regulating the response to MWCNTs. (**a**) Tissue-specific activity of *mir*-*35* in regulating the toxicity of MWCNTs in inducing intestinal ROS production. (**b**) Intestine-specific activity of *mir*-*35* in regulating the toxicity of MWCNTs in decreasing locomotion behavior. Prolonged exposure was performed from L1-larvae to adult day 1. MWCNT exposure concentration was 0.1 μg/L. Bars represent means ± SD. ^**^P < 0.01 vs control (if not specially indicated).

In nematodes, *mir*-*35* is expressed in some tissues, including intestine, muscle, neurons, and epidermis^20^. Mutation of *mir*-*35* induced a susceptibility to the toxicity of MWCNTs (0.1 μg/L) in inducing intestinal ROS production and in decreasing locomotion behavior (Fig. 1). Based on the rescue assays, we found that transgenic expression of neuronal, muscle, or epidermal *mir*-*35* did not obviously affect the susceptibility of *mir*-*35* mutant nematodes to the toxicity of MWCNTs (0.1 μg/L) in inducing intestinal ROS production (Fig. 1). In contrast, intestinal expression of *mir*-*35* could effectively suppress the susceptibility of *mir*-*35* mutant nematodes to the toxicity of MWCNTs (0.1 μg/L) in inducing intestinal ROS production and in decreasing locomotion behavior (Fig. 1). Therefore, *mir*-*35* can act in the intestine to regulate the response of nematodes to MWCNTs.

### Transcriptomic changes induced by MWCNTs (0.1 μg/L)

**Figure 2:**
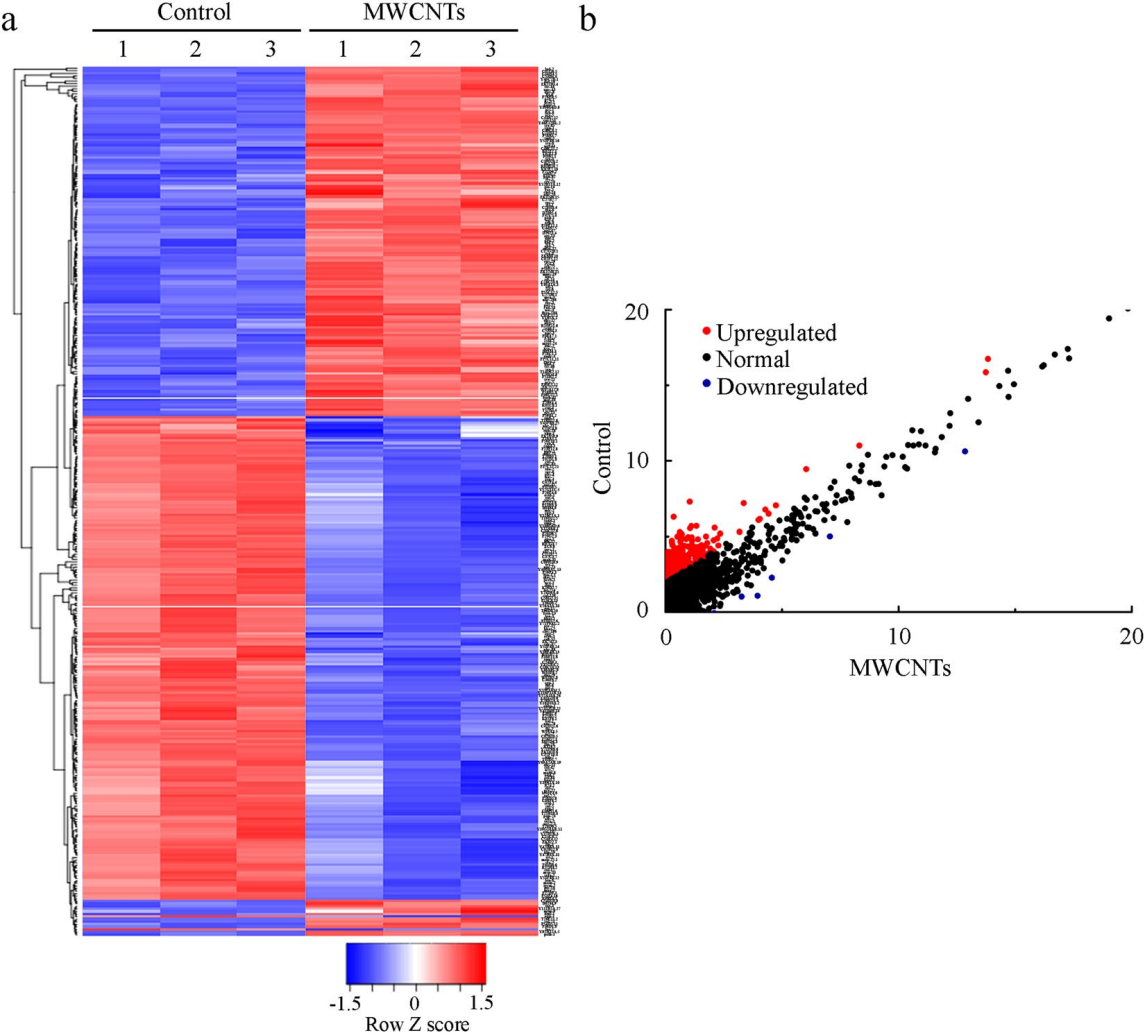
Dysregulated mRNAs induced by MWCNTs. (**a**) Heatmap showing the expression of mRNAs obtained from control and MWCNTs exposed nematodes. Relatively low expression levels are represented as blue, and relatively high expression levels are represented in red. (**b**) Scatter diagram of relationship between mRNA coverage of the control group and the MWCNTs exposure group. Prolonged exposure was performed from L1-larvae to adult day 1. MWCNT exposure concentration was 0.1 μg/L.

To identify the direct potential target(s) of *mir*-*35* in regulating the response to MWCNTs, we next determined the dysregulated gene profiling by MWCNTs (0.1 μg/L) using Illumina HiSeq^TM^ 2000 sequencing technique. Totally 342 differentially expressed mRNAs were identified in MWCNTs (0.1 μg/L) exposed nematodes compared with control (Fig. 2, Table S1). Among these 342 candidate mRNAs, 149 mRNAs were up-regulated and 193 mRNAs were down-regulated in MWCNTs (0.1 μg/L) exposed nematodes (Fig. 2, Table S1).

### Prediction of potential targets of *mir*-*35* in regulating the response to MWCNTs (0.1 μg/L)

**Figure 3:**
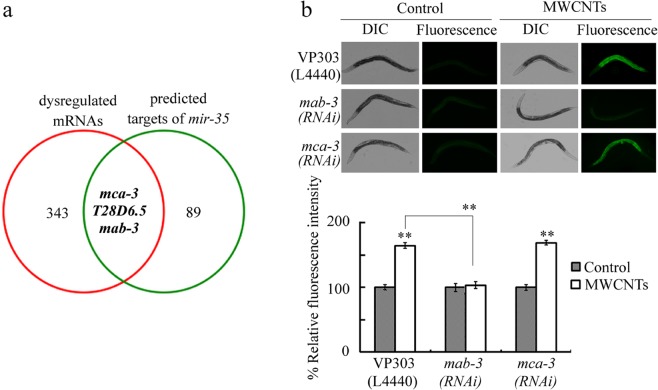
Identificatio of potential targets of *mir*-*35* in regulating the response to MWCNTs. (**a**) Search for the candidate targets for *mir*-*35* in regulating the response to MWCNTs. (**b**) Effect of intestine-specific RNAi knockdown of *mab*-*3* or *mca*-*3* on toxicity of MWCNTs in inducing intestinal ROS production. Prolonged exposure was performed from L1-larvae to adult day 1. MWCNT exposure concentration was 0.1 μg/L. Bars represent means ± SD. ^**^P < 0.01 vs control (if not specially indicated).

We next sought to identify the potential targets of *mir*-*35* during the control of response to MWCNTs. The corresponding targeted genes for *mir*-*35* were predicted using TargetScan by searching for the presence of conserved sites that match seed region of *mir*-*35* (version 6.2, http://www.targetscan.org/worm_52/) (Table S2). Among these predicted targeted genes, *mca*-*3*, *T28D6*.*5*, and *mab*-*3* could also be dysregulated by MWCNTs (0.1 μg/L) (Fig. 3a, Table S1).

Exposure to MWCNTs (0.1 μg/L) could decrease the expressions of *mab*-*3* and *mca*-*3*, and increase the expression of *T28D6*.*5* (Table S1). Considering the fact that exposure to MWCNTs (0.1 μg/L) could increase the *mir*-*35* expression (Fig. S1), we next focused on MAB-3 and MCA-3 to examine their role in regulating the response to MWCNTs. In nematodes, both MAB-3 and MCA-3 can be expressed in the intestine (https://wormbase.org). Using VP303 as a genetic tool, we found that intestine-specific RNA interference (RNAi) knockdown of *mca*-*3* did not obviously affect the toxicity of MWCNTs (0.1 μg/L) in inducing intestinal ROS production (Fig. 3b). Different from this, intestine-specific RNAi knockdown of *mab*-*3* significantly inhibited the toxicity of MWCNTs (0.1 μg/L) in inducing intestinal ROS production (Fig. 3b). That is, intestine-specific RNAi knockdown of *mab*-*3* induced a resistance to the MWCNT toxicity, implying that MAB-3 may act as a target for *mir*-*35* in regulating the response to MWCNTs. MAB-3 is a transcription factor in nematodes.

### *In vivo* 3′-untranslated region (3′-UTR) binding assay of *mir*-*35* with MAB-3

**Figure 4:**
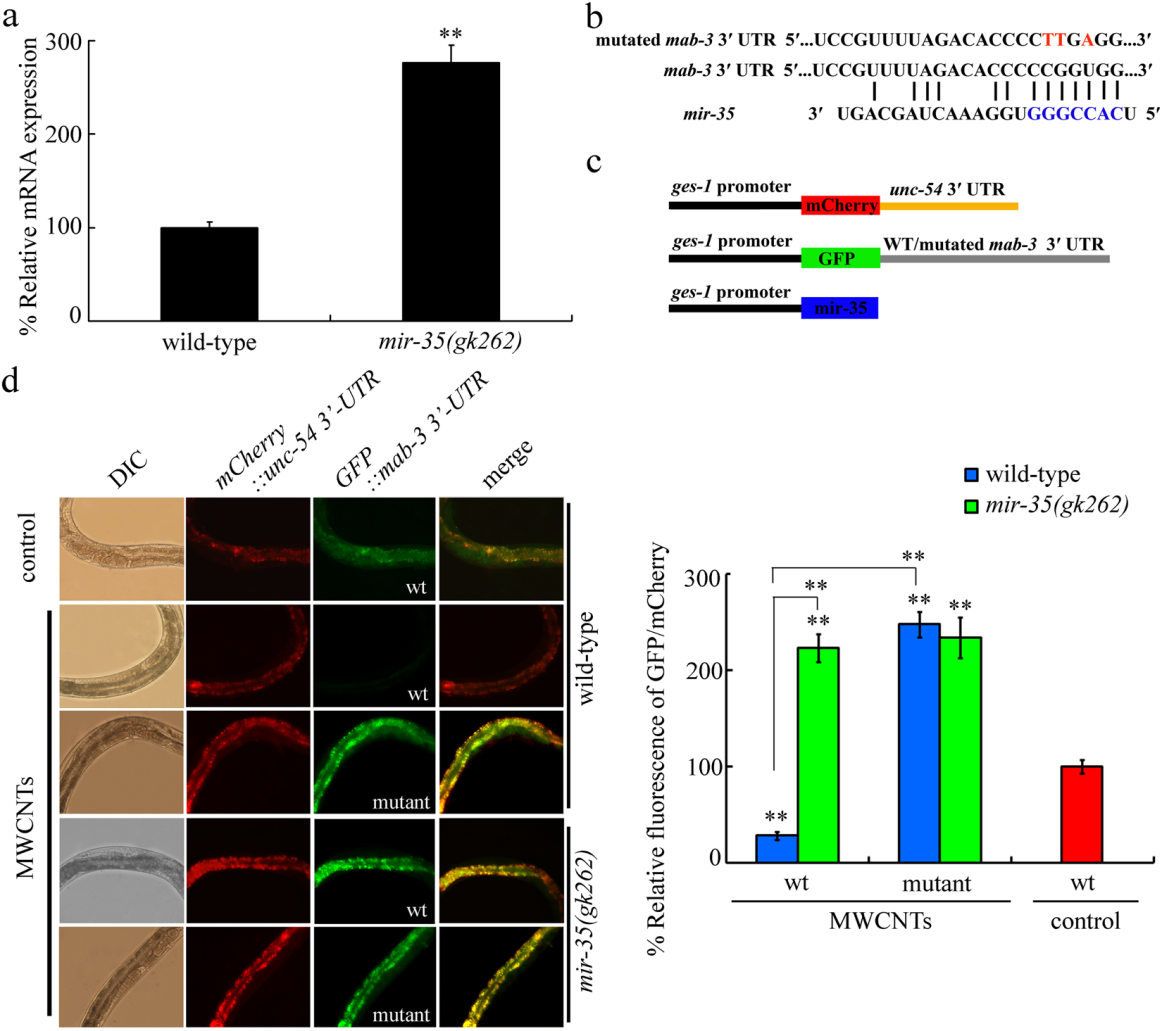
*In vivo* 3′-UTR binding assay of *mab*-*3*. (**a**) Effect of *mir*-*35* mutation on *mab*-*3* expression. Bars represent means ± SD. ^**^P < 0.01 vs wild-type. (**b**) Predicted binding site on *mab*-*3* 3′ UTR by Targetscan. *mir*-*35* seed sequence is shown in blue. (**c**) DNA construct outline. (**d**) Fluorescence images of the *mab*-*3 3*′-*UTR GFP* reporter in nematodes exposed to MWCNTs. wt, wild-type. MWCNT exposure concentration was 0.1 μg/L. Bars represent means ± SD. ^**^P < 0.01 vs wt/control (if not specially indicated).

In nematodes, we observed that loss-of-function mutation of *mir*-*35* could significantly increase the *mab*-*3* expression (Fig. 4a). To confirm whether MAB-3 is a direct target of *mir*-*35* during the regulation of response to MWCNTs, we constructed GFP vector driven by *ges*-*1* promoter, which contained 3′-UTR of *mab*-*3* (P*ges*-*1*::*GFP*-*3*′-*UTR (mab*-*3 wt)* or P*ges*-*1*::*GFP*-*3*′-*UTR (mab*-*3mutant)*. The *mir*-*35* binding site in *mab*-*3* 3′ UTR was mutated from CCCGGUG to CCTTGAG in order to prevent the binding of *mir*-*35* (Fig. 4b). The related vector contruction information was outlined in Fig. 4c. Considering the fact that the *mir*-*35* can not bind to *unc*-*54* 3′-UTR, a P*ges*-*1*::*mCherry*-*3*′-*UTR (unc*-*54)* was employed as an internal control. After MWCMT (0.1 μg/L) exposure, the GFP expression was significantly decreased in wild-type nematodes (Fig. 4d). In contrast, mutagenesis of binding site for *mir*-*35* in *mab*-*3* 3′-UTR abolished this GFP expression decrease in wild-type nematodes (Fig. 4d). After MWCNT (0.1 μg/L) exposure, a higher GFP expression was observed in *mir*-*35* mutant nematodes than that in wild-type nematodes (Fig. 4d). These observations support the role of MAB-3 as the direct target of intestinal *mir*-*35* during the control of response to MWCMTs in nematodes.

### Genetic interaction between *mir*-*35* and MAB-3 in regulating the response to MWCNTs

**Figure 5:**
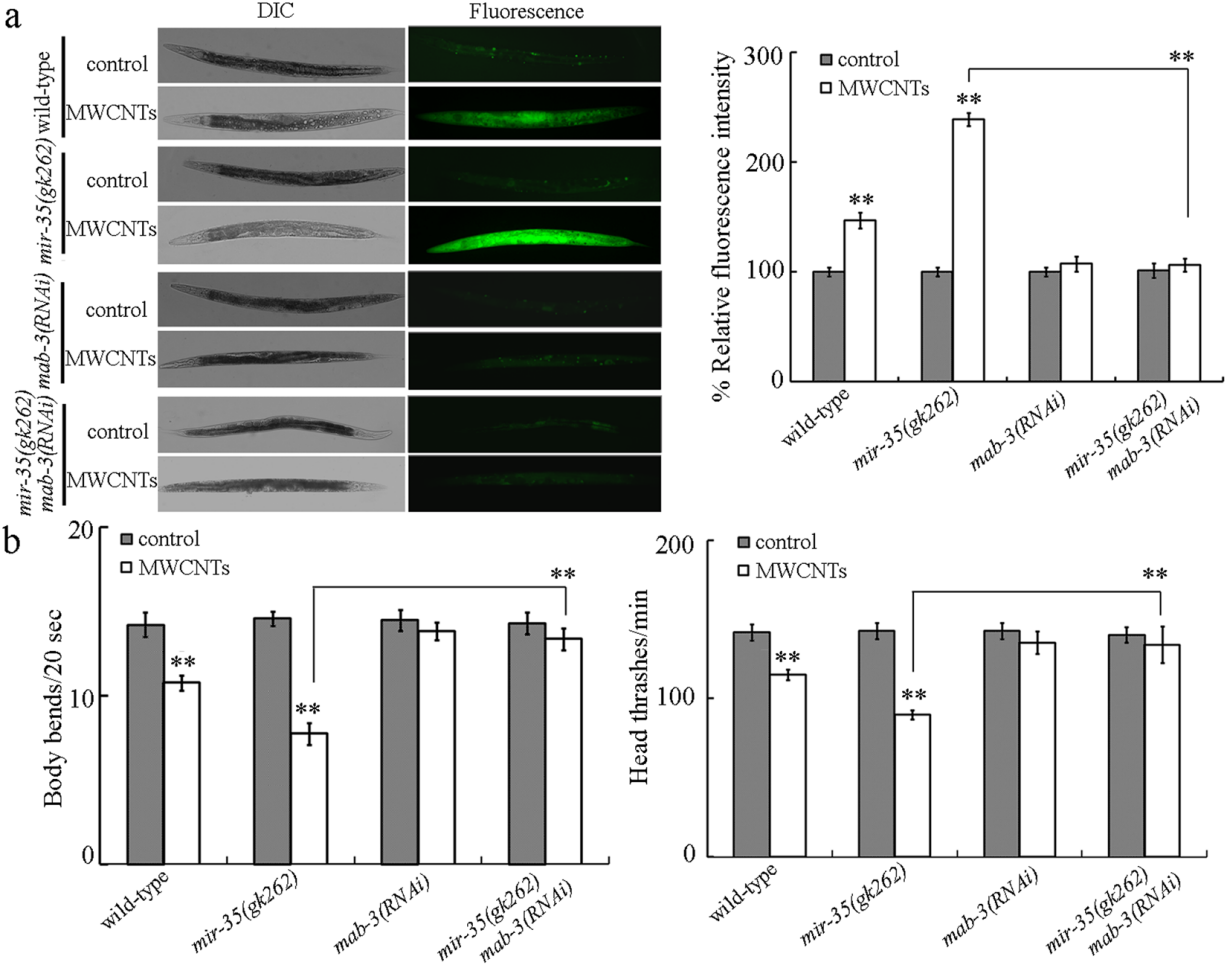
Genetic interaction between *mir*-*35* and MAB-3 in regulating the response to MWCNTs. (**a**) Genetic interaction between *mir*-*35* and MAB-3 in regulating the MWCNTs toxicity in inducing intestinal ROS production. (**b**) Genetic interaction between *mir*-*35* and MAB-3 in regulating the MWCNTs toxicity in decreasing locomotion behavior. MWCNT exposure concentration was 0.1 μg/L. Bars represent means ± SD. ^**^P < 0.01 vs control (if not specially indicated).

To further confirm the role of MAB-3 as the target of *mir*-*35* in regulating the response to MWCNTs, we investigated the genetic interaction between *mir*-*35* and MAB-3. We observed that both the intestinal ROS production and the locomotion behavior in MWCNTs exposed *mir*-*35(gk262)mab*-*3(RNAi)* nematodes were similar to those in MWCNTs exposed *mab*-*3(RNAi)* nematodes (Fig. 5). That is, RNAi knockdown of *mab*-*3* could effectively suppress the susceptibility of *mir*-*35* mutant nematodes to the MWCNTs toxicity.

### Genetic interaction between DAF-16 and MAB-3 in regulating the response to MWCNTs

**Figure 6:**
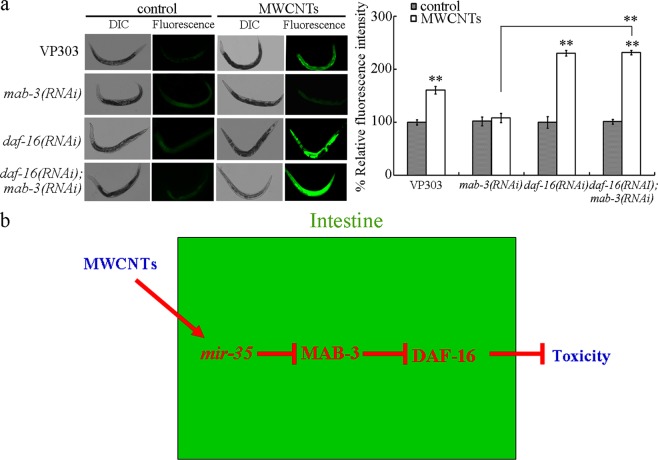
Genetic interaction between DAF-16 and MAB-3 in the intestine to regulate the response to MWCNTs. (**a**) Genetic interaction between DAF-16 and MAB-3 in the intestine to regulate the MWCNTs toxicity in inducing intestinal ROS production. MWCNT exposure concentration was 0.1 μg/L. Bars represent means ± SD. ^**^P < 0.01 vs control (if not specially indicated). (**b**) A diagram showing the mechanism for intestinal *mir*-*35* in regulating the response to MWCNTs in nematodes.

Our previous study has indicated that the insulin signaling pathway regulates the toxicity of MWCNTs in nematodes^21^. In the insulin signaling pathway, *daf*-*16* encodes a fork head transcriptional factor. Intestinal RNAi knockdown of *daf*-*16* induced a susceptibility to the MWCNTs toxicity in inducing intestinal ROS production and in decreasing locomotion behavior (Fig. 6a). Moreover, we found that intestinal RNAi knockdown of *daf*-*16* could further suppress the resistance of *mab*-*3(RNAi)* nematodes to the MWCNTs toxicity in inducing intestinal ROS production and in decreasing locomotion behavior (Fig. 6a). Therefore, MAB-3 may acts upstream of DAF-16 to regulate the response to MWCNTs in nematodes.

## Discussion

In this study, we observed that prolonged exposure (from L1-larvae to adult day-1) to MWCNTs (≥100 ng/L) could significantly increase the expression of *mir*-*35* (Fig. S1). Early in 2009, it was predicted that the environmentally relevant concentrations for CNTs are 6.6–31.5 ng/L for sewage treatment plant effluent^22^. With the rapid increase in production and in application of CNTs^23^, 100 ng/L can be or will be considered as the environmentally relevant concentration. Thus, long-term exposure to MWCNTs at environmentally relevant concentration may induce a *mir*-*35*-mediated response in nematodes.

In nematodes, *mir*-*35* is expressed in many tissues^20^. Meanwhile, we observed that the increase in *mir*-*35* mediated a protective response to MWCNTs^18^. Among the examined tissues, we found that *mir*-*35* only acted in the intestine to regulate the response to MWCNTs (Fig. 1). Therefore, the increase in *mir*-*35* only mediated an intestinal response of nematodes to MWCNTs. In the intestine, it was reported that the *mir*-*35* may also regulate the intestinal cell G1/S transition, since loss of *mir*-*35* leaded to a decrease of nuclei numbers in intestine of nematodes^24^. Besides this, it was also found that the *mir*-*35* could further regulate the germ cell proliferation or apoptosis by antagonizing certain molecular signal pathways, such as MAPK and core apoptosis pathways^24,25^, which suggests the germline activity of *mir*-*35* in regulating the other aspects of biological processes in nematodes.

To understanding the molecular mechanism for intestinal *mir*-*35* in regulating the response to MWCNTs, we tried to identify the potential target of intestinal *mir*-*35* during the control of response to MWCNT exposure. We raised several lines of evidence to prove the role of a DM domain transcription factor MAB-3 as the target of intestinal *mir*-*35* in regulating the response to MWCNTs. Firstly, loss-of-function of *mir*-*35* significantly increased the *mab*-*3* expression (Fig. 4a). Secondly, the phenotype of MWCNTs exposed *mab*-*3(RNAi)* nematodes was opposite to that in *mir*-*35* mutant nematodes (Fig. 3b). Previous study also suggested that RNAi knockdown of *mab*-*3* induced a resistance to oxidative stress^26^. Thirdly, 3′-UTR binding assay suggested the potential binding of intestinal *mir*-*35* with 3′-UTR of *mab*-*3* (Fig. 4d). Finally, functional analysis indicated that RNAi knockdown of *mab*-*3* could suppress the susceptibility of *mir*-*35* mutant nematodes to the MWCNTs toxicity (Fig. 5).

Insulin signaling pathway plays a crucial role in regulating the response of nematodes to various environmental toxicants or stresses^27^. In the insulin signaling pathway, the DAF-16 is a FOXO transcription factor, and DAF-16 usually act in the intestine to regulate the response of nematodes to various environmental toxicants or stresses by activating or inhibiting some of its downstream targets^27,28^. For the underlying of intestinal MAB-3 in regulating the response to MWCNTs, we found that intestinal RNAi knockdown of *daf*-*16* could inhibit the resistance of *mab*-*3(RNAi)* nematodes to MWCNTs toxicity (Fig. 6a). Therefore, intestinal MAB-3 may regulate the response to MWCNTs by further suppressing the function of DAF-16 and its downstream targeted genes in the insulin signaling pathway. So far, the downstream targeted genes for intestinal DAF-16 in regulating the response to MWCNTs are still unknown.

In this study, we employed *C*. *elegans* to determine the molecular basis for the increase in *mir*-*35* expression-mediated protective response to MWCNTs. The intestine-specific activity in regulating the response to MWCNTs was found in nematodes. In the intestine, a DM domain transcription factor MAB-3 acted as a target of *mir*-*35* during the control of response to MWCNTs (Fig. 6b). For the underlying mechanism, we found that intestinal MAB-3 regulated the response to MWCNTs by suppressing the function of DAF-16 in the insulin signaling pathway (Fig. 6b). The identified signaling cascade of *mir*-*35*-MAB-3-DAF-16 provides an important basis for intestinal response to environmental toxicants in nematodes.

## Methods

### MWCNTs properties

MWCNTs were from Shenzhen Nanotech Port Co. Ltd (Shenzhen, China). Working concentrations of MWCNTs were prepared by diluting the stock solution (1 mg/mL) with K-medium (50 mM NaCl, 30 mM KCl, and 10 mM NaOAc, pH 6.0). Before the use, the working solutions were sonicated for 30 min (40 kHz, 100 W). Based on the analysis of transmission electron microscopy (TEM) (JEM-200CX, JEOL, Japan), the diameter of MWCNTs was 10~20 nm, and the length of MWCNTs was 0.4~4 μm (Fig. S2). The zeta potential of MWCNTs was −32.4 ± 2.2 mV^18^. In the used MWCNTs, we detected the presence of 0.077% Ni and 0.017% Fe using elemental inductively coupled plasma mass spectrometry (ICP-MS) (Thermo Elemental X7, USA). Exposure from L1-larvae to adult day-1 to 0.077% Ni or 0.017% Fe did not induce the obvious ROS production and alteration in locomotion behavior (data not shown), suggesting the observed MWCNTs toxicity was not due to the impurity.

### Strain maintenance and exposure

Some of the used strains in the present study were originally obtained from Caenorhabditis Genetics Center. The used strains contain wild-type N2, mutant of *mir*-*35(gk262)*, and transgenic strains of *mir*-*35(gk262)Is(*P*ges*-*1*-*mir*-*35)*, *mir*-*35(gk262)Is(*P*unc*-*14*-*mir*-*35)*, *mir*-*35(gk262)Is(*P*myo*-*3*-*mir*-*35)*, *mir*-*35(gk262)Is(*P*mlt*-*7*-*mir*-*35)* and VP303/*rde*-*1(ne219); kbIs7*. VP303 is a genetic tool for intestine-specific RNAi knockdown of certain gene(s)^29^. Nematodes were maintained on nematode growth medium (NGM) plates seeded with *Escherichia coli* OP50 as food as described^30^. The collected gravid animals were first lysed using bleaching mixture solution (0.45 M NaOH, 2% HOCl). After that, the released eggs were used to prepare age synchronous L1-larvae.

Prolonged exposure to MWCNTs was performed from L1-larvae to adult day-1 in liquid solutions with the addition of OP50 (~4 × 10^6^ colony-forming units (CFUs)). The MWCNTs solutions were refreshed daily.

### Quantitative real-time polymerase chain reaction (qRT-PCR)

The animals were spun down in an eppendorf tube, and the total RNA extraction was performed with Trizol (Invitrogen, Carlsbad, CA). The cDNAs were synthesized by reverse transcription with the oligo-dT primer on total RNA. Quantitative PCR of target genes was carried out using SYBR® Green FastMix® according to manufacturer instruction with the ABI Prism7000a platform (Applied BioSystems, Warrington, UK) and normalized with the reference gene *tba*-*1* encoding a Tubulin protein. Primers used for qRT-PCR are listed in Table S3. The *mir*-*35* expression was expressed as relative expression ratio between *mir*-*35* and *F35C11*.*9* encoding a small nuclear RNA U6. Primer for reverse transcription of *mir*-*35* is GTCGTATCCAGTGCAGGGTCCGAGGTATTCGCACTGGATACGACACTGCTA. Primer for real-time PCR of *mir*-*35* is ATAATCTCACCGGGTGGAAACT, and common reward primer is GTGCAGGGTCCGAGGT. Forward primer *F35C11*.*9* is GAAGATTAGCATGAACCC, and reverse primer *F35C11*.*9* is TTGGAACGCTTTATGAAT. All reactions were performed in triplicate.

### Toxicity assessment

ROS production was used to reflect the activation of oxidative stress^31^. The method for detecting intestinal ROS production was performed as described^32^. The test nematodes were washed off the plates with K buffer, and incubated with freshly prepared 1 µM CM-H_2_DCFDA for 3 h in the dark. After that, the nematodes were mounted on agar pads for examination with a laser scanning confocal microscope (Ex: 480 nm; Em: 510 nm). The fluorescence intensities were examined by Image J (NIH), and the semi-quantified ROS was expressed as relative fluorescent units (RFU). For each treatment, fifty nematodes were examined.

Locomotion behavior was used to reflect the functional state of motor neurons^33^. Head thrash and body bend were used to reflect the locomotion behavior as described^34^. After MWCNTs exposure, the nematodes were transferred onto freshly made NGM plate without food. A change for bending direction at body mid-region of nematodes is considered as a head thrash, and a change of posterior bulb direction is considered as a body bend. For each treatment, fifty nematodes were examined.

### RNA-seq library preparation and HiSeq 2000 sequencing

HiSeq 2000 sequencing was performed as described previously^21^. MWCNT exposure concentration was 0.1 μg/L. After quality determination of RNA isolated using Nano Photometer P-Class, mRNA libraries were prepared with RNA-seq Sample Preparation kit (Illumina, Inc., San Diego, CA, USA) for the next Illumina HiSeqTM 2000 sequencing. Quality of reads was checked using Fast QC, and the total read numbers of control or MWCNTs exposure group data sets were normalized to equal levels. We determined dysregulated mRNAs in MWCNT (0.1 μg/L) exposed nematodes with fold change analysis together with the analysis based on statistical significance and use of a 2.0-fold change cutoff.

### RNAi assay

RNAi was performed by feeding animals with *E*. *coli* HT115 expressing double-stranded RNA corresponding to certain gene(s) as described^35^. The prepared L1-larvae were grown on RNAi plates. When they developed into gravid, the adult nematodes were transferred onto a fresh plate to obtain the second generation for the toxicity assessment. HT115 bacteria harboring empty vector L4440 containing two T7 promoters flanking a polylinker was used as a control. RNAi efficiency was confirmed by qRT-PCR (data not shown).

### DNA constructs and transformation

The promoter of *ges*-*1* (expressed in intestine), *unc*-*14* (expressed in neurons), *myo*-*3* (expressed in muscle), or *mlt*-*7* (expressed in epidermis) was amplified by PCR from wild-type genomic DNA. PCR amplified *mir*-*35* was inserted into vector pPD_95_77 carrying *ges*-*1*, *unc*-*14*, *myo*-*3*, or *mlt*-*7* promoter. Germline transformation was conducted by coinjecting a testing DNA (10–40 μg/mL) and a marker DNA of *Pdop*-*1::rfp* (60 μg/mL) into gonad of animals^36^. Primer information for vector constructions is shown in Table S4.

### 3′-UTR reporters and microscopy

The 3′-UTR (wt) of *mab*-*3* was PCR amplified from genomic DNA. A *mab*-*3* 3′-UTR (mutant) reporter was constructed by replacing *mir*-*35* binding site with an oligonucleotide containing complementary sequence of *mir*-*35*. The 3′ UTR reporter construct and mCherry internal control (P*ges*-*1*::*mCherry*-*3*′*UTR (unc*-*54)*) plasmid were coinjected into the gonad of nematodes as described^36^. GFP and mCherry expressions were analyzed under a fluorescence microscope. Related primer information for vector constructions is shown in Table S4.

### Statistical analysis

Statistical analyses were performed using SPSS 20.0 software (SPSS Inc., Chicago, USA). Differences between two groups were analyzed by student *t* test. Differences among more than two groups were analyzed by analysis of variance (ANOVA) and Dunnet’s test. Probability levels of 0.05 (^*^) and 0.01 (^**^) were considered to be statistically significant.

## Supplementary information

Supporting Information
