# Supplementary material for: Prolonged exposure to multi-walled carbon nanotubes dysregulates intestinal mir-35 and its direct target MAB-3 in nematode Caenorhabditis elegans
Source: Sci Rep. 2019 Aug 21;9:12144. doi: 10.1038/s41598-019-48646-8 (PMC6704117; doi:10.1038/s41598-019-48646-8)
Supplement: Supplementary file 1 — Supporting Information [file 41598_2019_48646_MOESM1_ESM.pdf]

Prolonged exposure to multi-walled carbon nanotubes dysregulates intestinal *mir-35* and its direct target MAB-3 in nematode *Caenorhabditis elegans*

Yunli Zhao[1, 2, *], Ling Jin[1], Yuan Wang[1], Yan Kong[2] & Dayong Wang[2, *]

[1]Department of Preventive Medicine, Bengbu Medical College, Bengbu 233030, China

[2]Medical School, Southeast University, Nanjing 210009, China

[*]Correspondence and requests for materials should be addressed to Y.Z (yunli201@126.com) and D.W. (dayongw@seu.edu).

Supporting Information:

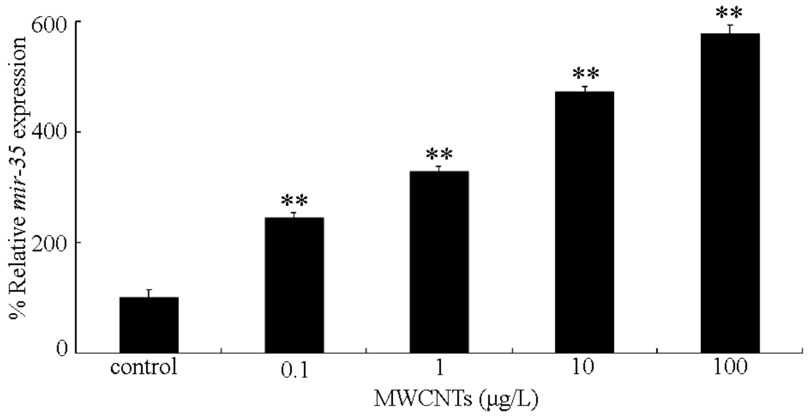

Figure S1. Effect of MWCNTs exposure on *mir-35* expression. Bars represent means ± SD. $^{**}P < 0.01$ *vs* control.

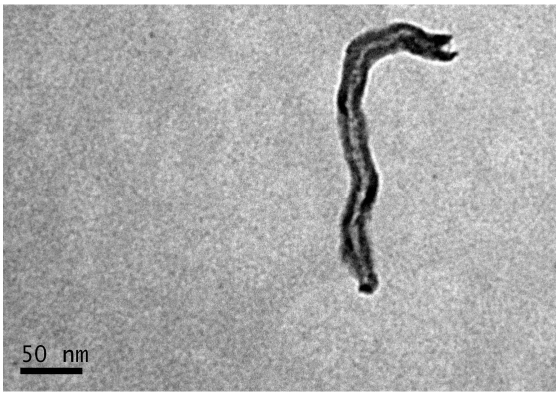

Figure S2. TEM of MWCNTs in K buffer after sonication.

Table S1. Dysregulated mRNAs induced by MWCNTs (0.1 μg/L)

Gene ID	FC	*P* Value	Regulation	Gene ID	FC	*P* Value	Regulation
lad-2	-5.75	4.10E-18	down	*npr-23*	2	2.02E-03	up
C01G6.5	-5.48	4.03E-10	down	*Y48G8AL.13*	2.01	4.80E-04	up
C34H4.5	-5.19	4.33E-10	down	*F59F4.3*	2.04	1.21E-03	up
C53B4.4	-4.88	9.36E-08	down	*fpn-1.1*	2.04	3.77E-04	up
Y40C7B.1	-4.83	6.49E-12	down	*mec-17*	2.04	9.46E-04	up
daf-19	-4.71	4.21E-08	down	*pccb-1*	2.06	3.16E-05	up
ZK1193.4	-4.71	3.61E-10	down	*ncx-1*	2.07	6.18E-05	up
ttr-44	-4.68	2.77E-11	down	*dpt-1*	2.08	1.51E-04	up
unc-43	-4.59	8.52E-07	down	*K10D2.7*	2.08	6.89E-04	up
dpy-8	-4.59	8.52E-07	down	*let-754*	2.09	2.20E-03	up
lst-6	-4.45	2.38E-06	down	*Y24D9A.6*	2.09	2.99E-03	up
F26H9.5	-4.44	4.34E-07	down	*col-138*	2.1	1.08E-03	up
pyk-1	-4.43	9.10E-13	down	*C56G2.15*	2.1	9.78E-05	up
dcaf-1	-4.26	2.15E-09	down	*F23C8.13*	2.11	2.19E-05	up
Paqr-1	-4.22	2.42E-06	down	*T06D8.2*	2.11	9.46E-04	up
Y106G6D.8	-4.21	2.58E-15	down	*Y56A3A.33*	2.12	7.22E-05	up
acs-1	-4.13	2.21E-05	down	*gei-15*	2.14	6.35E-05	up
fkb-6	-4.12	3.23E-11	down	*T06D8.10*	2.15	2.12E-03	up
acr-5	-4.04	0.000259	down	*lron-13*	2.15	8.25E-04	up
C44B7.12	-4.00	1.12E-08	down	*lon-3*	2.16	3.76E-03	up
col-153	-3.99	5.72E-07	down	*hhat-1*	2.16	1.95E-04	up
Y46E12BL.2	-3.94	0.000555	down	*F13D12.6*	2.21	4.84E-05	up
srz-85	-3.94	0.000555	down	*Y71F9AL.2*	2.22	9.40E-04	up
rpn-1	-3.91	3.20E-07	down	*avr-15*	2.23	4.25E-03	up
C49C8.2	-3.86	4.46E-08	down	*hhat-2*	2.23	4.86E-04	up
C23H4.2	-3.83	0.000821	down	*clec-196*	2.24	1.08E-03	up
F58B3.7	-3.83	0.000821	down	*epg-2*	2.24	1.43E-04	up

smk-1	-3.83	0.000821	down	*ergo-1*	2.25	7.12E-04	up
Y53F4B.16	-3.83	0.000821	down	*ceh-51*	2.25	3.47E-03	up
srg-8	-3.78	1.52E-06	down	*ZK742.3*	2.25	4.63E-05	up
ugt-44	-3.75	6.11E-05	down	*cdc-37*	2.26	1.08E-03	up
C46C11.2	-3.72	0.001222	down	*Y53F4B.14*	2.28	2.83E-03	up
R02F2.1	-3.72	0.001222	down	*nhr-88*	2.28	2.71E-04	up
T02E1.8	-3.72	0.001222	down	*Y39E4B.13*	2.3	3.95E-05	up
PDB1.1	-3.72	0.001222	down	*W05H7.1*	2.3	2.43E-04	up
srz-3	-3.72	0.001222	down	*F38E11.6*	2.33	1.52E-03	up
C39D10.2	-3.71	2.37E-05	down	*vnut-1*	2.33	1.52E-03	up
dnj-16	-3.71	1.53E-06	down	*C24B9.3*	2.33	1.76E-06	up
R03H10.7	-3.67	8.69E-05	down	*Y42G9A.2*	2.37	1.89E-03	up
K02E7.10	-3.65	4.30E-05	down	*C16C10.13*	2.38	4.55E-03	up
F22D6.2	-3.59	0.002768	down	*Y48A6C.6*	2.38	4.99E-05	up
pdhk-2	-3.59	0.002768	down	*W03F9.2*	2.42	1.27E-03	up
pqn-92	-3.59	0.002768	down	*dnj-29*	2.46	8.73E-05	up
lys-5	-3.59	0.002768	down	*W02H5.8*	2.46	4.92E-04	up
syx-18	-3.57	7.51E-12	down	*C36E6.2*	2.48	2.91E-06	up
Y52B11A.12	-3.53	6.43E-06	down	*cgp-1*	2.49	5.99E-05	up
gei-14	-3.52	1.00E-05	down	*fut-6*	2.5	5.33E-04	up
rer-1	-3.51	1.64E-08	down	*pir-1*	2.52	5.56E-05	up
chil-14	-3.48	8.25E-06	down	*Y55F3AM.5*	2.53	1.38E-04	up
nhr-20	-3.45	0.004213	down	*Y116F11B.14*	2.53	1.38E-04	up
ZK1248.15	-3.45	0.004213	down	*Y57A10A.26*	2.53	9.02E-05	up
C27B7.7	-3.45	0.004213	down	*C01G10.9*	2.55	3.79E-04	up
spc-1	-3.44	0.000149	down	*Y79H2A.4*	2.58	3.20E-04	up
scl-1	-3.44	0.000149	down	*Y18D10A.2*	2.59	4.80E-04	up
C28H8.4	-3.44	0.000149	down	*ehbp-1*	2.59	4.80E-04	up
tba-6	-3.44	0.000149	down	*Y71G12B.25*	2.59	6.61E-05	up

D2005.4	-3.42	4.38E-08	down	*Y41D4B.14*	2.59	1.55E-03	up
F42C5.4	-3.39	0.000378	down	*F39B2.9*	2.6	3.18E-03	up
pah-1	-3.36	2.20E-09	down	*F25B5.5*	2.63	3.97E-04	up
egl-4	-3.34	4.30E-05	down	*K02F6.1*	2.63	3.97E-04	up
ech-8	-3.30	5.37E-06	down	*kel-1*	2.63	2.29E-04	up
F59E12.1	-3.29	0.000821	down	*unc-76*	2.63	3.18E-03	up
C44B7.5	-3.29	0.000821	down	*C41D11.6*	2.65	1.02E-05	up
ugt-47	-3.29	0.000821	down	*flp-2*	2.66	1.26E-04	up
D2023.4	-3.29	0.000821	down	*W04A4.5*	2.66	1.02E-03	up
atg-13	-3.22	1.84E-09	down	*ncs-2*	2.66	1.02E-03	up
dpf-1	-3.19	0.00078	down	*C02B10.5*	2.67	2.49E-03	up
pgp-2	-3.17	0.001222	down	*C56G7.3*	2.68	6.52E-06	up
tre-1	-3.17	0.001222	down	*M05D6.3*	2.68	2.85E-05	up
gbb-2	-3.11	3.57E-07	down	*flp-28*	2.69	7.48E-07	up
mltn-12	-3.10	0.001103	down	*R119.5*	2.71	8.27E-04	up
C17G10.1	-3.10	0.001103	down	*Y71F9B.6*	2.73	1.94E-03	up
tra-4	-3.10	0.001103	down	*ZK1010.6*	2.76	1.52E-03	up
ZK688.10	-3.05	0.002768	down	*C32F10.4*	2.77	6.07E-06	up
C02F5.12	-3.05	0.002768	down	*nlp-2*	2.8	4.52E-03	up
vps-29	-3.02	1.19E-07	down	*T01D1.7*	2.81	1.20E-03	up
cysl-3	-3.01	2.36E-08	down	*Y69A2AR.19*	2.82	1.26E-06	up
srh-75	-3.01	1.21E-05	down	*dhs-11*	2.83	1.76E-04	up
F20D12.2	-3.01	1.79E-06	down	*col-47*	2.83	5.36E-05	up
ZK1248.11	-3.00	0.001567	down	*nsy-7*	2.84	3.36E-03	up
mms-19	-3.00	0.001567	down	*madf-8*	2.84	3.36E-03	up
lipl-1	-2.99	6.79E-05	down	*add-2*	2.84	3.36E-03	up
ttr-14	-2.96	4.85E-08	down	*col-84*	2.84	9.42E-04	up
C16C10.8	-2.94	0.00038	down	*bgal-1*	2.85	8.51E-06	up
Y40A1A.3	-2.94	0.00038	down	*Y39A1A.10*	2.87	7.43E-04	up

ufl-1	-2.94	1.98E-05	down	*R74.2*	2.88	1.24E-04	up
set-6	-2.92	9.48E-05	down	*ekl-7*	2.88	3.36E-03	up
F55C12.5	-2.91	0.004213	down	*gcy-22*	2.89	1.15E-07	up
C27D9.1	-2.91	0.004213	down	*M03F4.6*	2.9	1.07E-08	up
mca-3	-2.91	0.004213	down	*taf-7.2*	2.92	2.51E-03	up
nhr-286	-2.91	0.004213	down	*F58D5.9*	2.92	2.51E-03	up
tbc-3	-2.91	0.004213	down	*C08F8.2*	2.95	4.65E-04	up
puf-12	-2.91	0.004213	down	*irld-1*	2.95	2.13E-05	up
rad-54	-2.89	0.003211	down	*snf-5*	2.96	5.52E-07	up
nos-3	-2.89	6.26E-07	down	*rog-1*	3.03	1.41E-03	up
fbxa-188	-2.88	7.54E-09	down	*C06H5.6*	3.1	6.60E-06	up
C05C12.1	-2.86	0.000462	down	*T21B10.4*	3.14	1.50E-04	up
Y1B5A.1	-2.81	5.07E-05	down	*pqn-74*	3.22	9.14E-07	up
dhs-17	-2.80	0.000155	down	*ads-1*	3.23	4.62E-04	up
abts-1	-2.80	0.000608	down	*vesa-1*	3.24	9.35E-06	up
R10E11.6	-2.79	1.33E-07	down	*ucr-2.3*	3.31	6.64E-07	up
scpl-1	-2.79	0.000695	down	*T28D6.5*	3.32	1.11E-06	up
C53B4.3	-2.79	0.000695	down	*Y39G10AR.11*	3.32	1.56E-07	up
unc-3	-2.79	0.001103	down	*col-107*	3.33	4.11E-05	up
F08A7.1	-2.78	0.004633	down	*Y17D7B.3*	3.35	5.31E-04	up
marc-3	-2.78	0.004633	down	*C15C8.7*	3.38	5.31E-04	up
cyld-1	-2.78	0.004633	down	*C54E4.12*	3.4	2.97E-07	up
mrps-24	-2.78	0.004633	down	*ZK822.1*	3.42	3.70E-09	up
dnc-2	-2.77	2.91E-05	down	*flp-3*	3.46	6.23E-07	up
hsp-17	-2.76	1.63E-07	down	*Y47G6A.31*	3.52	4.66E-03	up
B0334.3	-2.75	5.22E-08	down	*C01B12.8*	3.52	4.66E-03	up
F28C1.1	-2.74	7.18E-07	down	*klp-19*	3.54	1.27E-08	up
gadr-3	-2.73	0.000802	down	*Y47D3A.31*	3.57	6.71E-06	up
F17C11.11	-2.71	0.000122	down	*ist-1*	3.57	4.66E-03	up

gpcp-1	-2.65	0.00141	down	*mop-25.1*	3.57	4.66E-03	up
pho-1	-2.64	4.97E-07	down	*srv-2*	3.58	6.71E-06	up
str-46	-2.64	0.000413	down	*T03D8.6*	3.66	2.43E-03	up
srg-1	-2.62	0.001769	down	*K12H4.2*	3.66	2.43E-03	up
T14G12.12	-2.62	0.001769	down	*col-17*	3.69	7.65E-05	up
C30G4.4	-2.62	0.001769	down	*msp-33*	3.71	2.43E-03	up
F53H2.1	-2.62	0.001769	down	*srxa-1*	3.71	2.43E-03	up
sru-21	-2.62	1.45E-05	down	*Y53F4B.12*	3.72	5.48E-06	up
gcy-5	-2.62	1.45E-05	down	*lon-8*	3.75	1.77E-03	up
F35C5.12	-2.61	2.56E-05	down	*mam-2*	3.82	2.08E-09	up
ZK757.2	-2.61	0.000608	down	*igcm-2*	3.83	1.29E-03	up
W07A12.8	-2.60	1.24E-06	down	*ptr-18*	3.83	1.29E-03	up
F58H1.8	-2.57	0.000117	down	*max-2*	3.87	9.45E-04	up
F08G12.1	-2.56	2.63E-05	down	*F27D9.3*	3.94	6.95E-04	up
bath-41	-2.55	2.92E-06	down	*F53F4.16*	3.98	5.13E-04	up
mig-38	-2.55	9.00E-06	down	*K09E4.1*	4.03	5.52E-06	up
T28F4.4	-2.54	0.000802	down	*C54D10.8*	4.12	2.11E-04	up
K01C8.1	-2.54	7.49E-05	down	*B0244.9*	4.15	2.11E-04	up
cat-4	-2.53	0.003361	down	*toe-4*	4.3	6.79E-05	up
T12B3.4	-2.53	0.003361	down	*Y111B2A.27*	4.35	4.40E-07	up
gly-13	-2.53	0.003361	down	*mtm-9*	4.44	3.01E-05	up
F59B1.2	-2.53	0.003361	down	*dml-1*	4.44	3.01E-05	up
imb-1	-2.52	4.56E-05	down	*aqp-1*	4.46	2.31E-05	up
T19D12.6	-2.52	9.10E-05	down	*T26C11.2*	4.46	2.31E-05	up
Y43F8B.25	-2.51	0.000883	down	*col-73*	4.47	1.50E-07	up
nhr-217	-2.48	0.000109	down	*F54D5.11*	4.56	7.58E-08	up
C13C4.8	-2.48	1.12E-05	down	*F36D1.8*	4.66	6.43E-06	up
twk-28	-2.47	0.000465	down	*far-5*	4.69	3.93E-16	up
vhp-1	-2.47	0.00141	down	*Y92H12A.5*	5.93	3.84E-10	up

ZK1058.9	-2.45	2.13E-06	down	*polh-1*	6.25	1.48E-11	up
T19A5.1	-2.43	0.00466	down				
F56F10.1	-2.43	0.00466	down				
rab-8	-2.42	0.0003	down				
copd-1	-2.41	0.004519	down				
F23F12.8	-2.41	0.004519	down				
ppfr-1	-2.34	0.000212	down				
wdr-23	-2.33	0.000138	down				
T20B3.1	-2.32	4.42E-05	down				
T02D1.8	-2.31	0.003363	down				
cut-3	-2.30	0.003291	down				
clec-83	-2.29	2.44E-06	down				
F37C12.21	-2.29	9.67E-05	down				
tdo-2	-2.29	0.001544	down				
snr-6	-2.24	3.77E-06	down				
pbs-4	-2.24	4.58E-06	down				
nas-7	-2.23	0.001042	down				
zen-4	-2.23	3.00E-05	down				
C02F4.4	-2.23	0.001945	down				
oac-41	-2.22	0.004313	down				
F32D8.5	-2.18	1.25E-05	down				
Y57G11C.5	-2.17	0.000147	down				
F26E4.6	-2.17	0.003066	down				
ostd-1	-2.16	0.000185	down				
eat-4	-2.16	9.46E-06	down				
yars-1	-2.16	0.003185	down				
F21F3.6	-2.16	1.13E-05	down				
F13H8.9	-2.15	0.00036	down				
B0464.9	-2.15	0.001069	down				

mlc-2	-2.13	2.42E-05	down
cpg-3	-2.13	9.47E-05	down
Y119C1A.1	-2.10	0.003867	down
T19D12.5	-2.10	2.10E-05	down
sand-1	-2.05	0.000771	down
zmp-1	-2.05	2.47E-05	down
Y106G6D.6	-2.05	2.48E-05	down
mab-3	-2.04	2.48E-05	down
Y12A6A.1	-2.04	9.96E-05	down
C34C12.6	-2.04	0.000303	down
F20D6.5	-2.04	6.73E-05	down
F20G2.3	-2.02	0.002612	down
gpa-1	-2.02	0.002612	down
hlh-12	-2.02	0.002612	down
R07G3.7	-2.01	6.96E-05	down

Table S2. A list of potential direct targets for *mir-35*

Predicted targets	Intestinal expression	Predicted targets	Intestinal expression	Predicted targets	Intestinal expression
tag-310		*C39E9.8*		*cyd-1*	+
R05H11.2		*D2092.4*		*dnj-1*	
hlh-11	+	*F07C6.4*		*egl-13*	
msi-1		*F13H8.5*		*letm-1*	
gei-16		*F26A3.4*		*lin-23*	
nhl-2		*K03B8.6*	+	*mca-3*	+
F25H2.5	+	*T05C1.1*		*mig-10*	
T28D6.5		*T06D8.1*		*mrp-7*	+
F32D8.1		*T28D6.4*		*nlp-15*	
par-1		*W05B5.1*		*rgs-6*	
F58E10.1		*Y11D7A.14*		*snf-1*	
dab-1	+	*Y44A6D.3*		*tnt-2*	
Y24D9A.1	+	*ZK899.1*	+	*uig-1*	
Y55F3AM.10		*lin-2*		*unc-49*	
cab-1		*F09G2.1*	+	*unc-52*	
C13B4.1	+	*ser-3*	+	*trp-4*	
alr-1	+	*F29B9.8*		*ZK1321.2*	
ceh-18		*Y71F9B.8*		*C30F12.2*	+
egl-3	+	*lin-59*	+	*ptr-23*	
rig-6		*F13H6.1*		*dsh-1*	+
C04C11.2		*tsn-1*	+	*D1054.9*	
lin-29		*rig-4*	+	*bath-40*	
F53B1.3		*tag-275*		*nas-1*	
wrt-3		*toh-1*	+	*egl-1*	
C11E4.4		*C06E1.3*		*snb-2*	
ced-1	+	*Y67A10A.8*		*bli-4*	

cex-2	+	*F42H10.3*	*tsp-20*
cho-1		*mab-3*	*spn-4*
cutl-15		*ztf-11*	*uvt-2*
H10E21.5			

Table S3. Primer information for PCR

	Forward primer (5'-3')	Reverse primer (5'-3')
tba-1	TCAACACTGCCATCGCCGCC	TCCAAGCGAGACCAGGCTTCAG
mab-3	AAATCCCGAGATGGTAAA	GCCAGCGTCAGAGTGTAT

Table S4. Primer information for DNA constructs

	Forward primer (5'-3')	Reverse primer (5'-3')
Pges-1	GCGTCTAGACACCAATACCTTTAGT GAC	GGGGATCCCTGAATTCAAAGATAAGA TATGT
Punc-14	ACGAAGCTTTTCCCAACTGGCAATA CT	TACTGCAGCCACAAAAGTTGAGAGC A
Pmyo-3	GCGAAGCTTGCTCCGCTGACTTTAG	ATAGGATCCAGTGGTCGTGGGTTTG
Pmlt-7	CATCTGCAGGGCAGGTGCAGGTAA AATGAAGG	GCGTCTAGATGGAGTCTTCTCATGGT GTTCC
mir-35	GCGCCATGGTCTGGAAGAAAAAGT ATATATAG	CGCCTCGAGTGTTAGTTTTAAGTTTAA GTTTA
3' UTR mab-3^{wt}	GTCGGATCCAAGATCTATAATTTTG ACCAATT	GTACCATGGAAATGAGACAGAAATGG ATGGAA
3' UTR mab-3^{mu}	GTCGGATCCAAGATCTATAATTTTG ACCAATT	CTACCATGGCCTCAAGGGGTGTCTAA AACGGA
mab-3	AAATCCCGAGATGGTAAA	GCCAGCGTCAGAGTGTAT
